# Business intelligence model empowering SMEs to make better decisions and enhance their competitive advantage

**DOI:** 10.1007/s44257-022-00002-3

**Published:** 2023-02-06

**Authors:** Konstantina Ragazou, Ioannis Passas, Alexandros Garefalakis, Constantin Zopounidis

**Affiliations:** 1 Department of Accounting and Finance, University of Western Macedonia, Kila, 50100 Kozani, Greece; 2 Department of Business Administration, Neapolis University Pafos, 8042 Pafos, Cyprus; 3 Department of Business Administration and Tourism, Hellenic Mediterranean University, 71410 Heraklion, Greece; 4 Financial Engineering Laboratory, Technical University of Crete, 73100 Chania, Greece; 5 Dept. of Management, Audencia Business School, 44312 Nantes, France

**Keywords:** Business Intelligence Model, Competitive advantage, Decision—making process, Analytics, Emerging technologies, Efficiency, Business continuity

## Abstract

Small and medium-sized businesses (SMEs) typically steer clear of implementing business intelligence (BI) systems because they feel that this sort of modeling is complicated and costly. But the market for business intelligence (BI) has evolved quickly. New opportunities like cloud computing have greatly lowered prices and eventually made it possible to design integrated solutions that are only intended for SMEs. In addition to highlighting the research trends in the sector under investigation, this paper explores the function of business intelligence in enhancing the decision-making process and competitive advantage of SMEs. The research subject has been approached using bibliometric analysis using the R package. The display of the results was done with the aid of Biblioshiny and VOSviewer's bibliometric tools. The study highlighted that SMEs have started integrating Business Intelligence systems. However, a new business model that will combine business analytics and will ensure to SMEs that emerging technologies will not affect them negatively is crucial. Thus, this research proposes the development of a new business model that will be based on Business Intelligence and Technology–organization–environment framework (TOE) framework, which helps SMEs to feel safe with emerging technologies.

## Introduction

Business intelligence (BI) systems are becoming more popular and are used by both large and small and medium-sized businesses. They are utilized to support a variety of business decisions, from tactical to strategic ones. BI may be seen as a barrier that protects enterprises as well as a collection of tools and best practices that enable senior executives to access and analyze corporate data and transform it into information that can be used to make rapid choices. The display of the results was done with the help of Biblioshiny and VOSviewer’s bibliometric tools [1, 2]. In short, BI may be defined as the process that helps to highlight the most pertinent information from a vast amount of data. BI is not a product or technology that can be bought and placed in a business to address problems, even though many tools on the market can assist in the implementation of such systems. As a result, BI integrates products, technology, and processes to arrange the crucial data that management should use to boost the performance and revenues of the company [3, 4].

The first reference on the term of Business Intelligence was emerged in 1958 by the researcher Luhn H.P., who defined it as the ability to grasp the interrelationships of events, which present themselves in such a way as to guide their action towards a desired goal [5]. Several years later, in 1989 Howard Dresner defined Business Intelligence (BI) as a broad term that includes concepts and methods that improve the ability of businesses to make decisions through decision support systems based on recorded events. For Business Intelligence many definitions have been given from time to time by several authors and researchers. Pechenizkiy in 2006 defined it as the new technology for understanding the past and predicting the future. Although, Business Intelligence is a broad category of technology that enables the collection, storage, access, and analysis of data to help business executives make better decisions and analyze their business performance through deep insight into data. Business Intelligence as we know it today evolved from Decision Support Systems (DSS) that began in the 1960s and evolved in the mid-1980s, to lead in the late 1980s to Executive Support Systems Information Systems (ESS), in Management Information Systems (MIS), and later in data warehouses and Business Intelligence systems which replaced these older systems. European Union’s systems no longer offer simple static analyzes like previous systems but are a comprehensive solution for businesses and organizations for strategy planning, customer relationship management and collaboration [4, 5].

Tools of business intelligence speed up the information analysis and performance evaluation, but are valuable in helping companies to reduce inefficiencies, illustrate possible difficulties, indicate profits, to enhance more their competitive advantage and improve the decision-making process [5, 6]. SMEs that function as social communities exist to enhance competitive advantage by creating and using new ideas and knowledge, while some researchers focus on the application of knowledge and argue that SMEs are entities that must apply knowledge to enhance their competitive advantage [7, 8]. Moreover, the main function of SMEs, like any form of business, is “how to make better use of the knowledge that their employees possess but this knowledge is not transmitted accordingly within the organization”. However, business intelligence can contribute to the way knowledge is created by upgrading capabilities by incorporating emerging technologies and interacting with the external environment of firms [2, 6, 9].

The current research work aims: (i) to highlight the role of Business Intelligence in the enhancement of the competitive advantage of SMEs and the improvement of the decision-making process and (ii) to indicate the research trends of the studied field [10, 11]. To approach the research question, a bibliometric analysis has been applied. The data retrieved from Scopus and for the visualization of the findings the bibliometric tools of Biblioshiny and VOSviewer have been used.

There are five sections in the paper. The literature study on the topic of business intelligence and how it benefits SMEs is presented in “ Literature review ” section. The tools and techniques are listed in “ Materials and methods ” section. The results are illustrated in “ Results ” section, and the article is discussed and concluded in “ Discussion and conclusions ” section.

## Literature review

The information revolution is a fact that significantly affects competition, as it changes the structure of the industry and by extension affects the rules of competition. Furthermore, the exploitation of information can create a competitive advantage in a firm, as it enables it to stand out from its competitors. In this work, the benefits obtained by a small and medium-sized enterprise by joining the strategy that uses Business Intelligence systems are combined with the creation and maintenance of a competitive advantage [4].

The advancement of technology contributes to the storage of large volumes of data compared to the past. Thus, businesses can collect data from both customers and competing businesses, which helps them differentiate themselves. This data is considered as an asset, which depending on the capabilities of each company, can be exploited to create a competitive advantage. Information systems, including Business Intelligence, facilitate the execution of a company's strategy and strengthen it in such a way that it stands out in the industry in which it operates [12, 13]. In the modern business environment, knowledge management through systems that collect, and process information help them businesses to develop a competitive advantage.

Generally, information technology is fundamentally changing the way businesses operate. It affects the process by which firms produce their products and reshapes the product itself: the set of physical goods, services and information that firms provide to create value for buyers. Information technology has for several years facilitated the submission of past information and provides information that can be used for decision making in the present as well as for future planning. Business Intelligence systems help an organization by enabling the dissemination of real-time information in a user-friendly manner. This includes providing users with a single point of access to important information, using a dynamic classification system. Business Intelligence systems also contribute to the creation of new knowledge based on past information.

In any business, information technology plays an important role in creating competitive advantage, whether through cost leadership or differentiation. Technology affects the activities that create value for the firm or enable firms to gain a competitive advantage by exploiting changes in the competitive environment [14].

Business Intelligence can be the difference between a company with an average performance and a company with a higher performance. A business can gain a competitive advantage by using Business Intelligence systems to align its strategic goals with its business initiatives [15]. Gaining a competitive advantage is crucial for a business. Competitive advantage has been defined as a product or service that a firm’s customers value more highly than comparable products or services of competitors [4]. In short, a business has a competitive advantage when it provides something useful (a product, service, or skill) that other businesses do not. Competitive advantages are usually temporary, as competitors are constantly looking for ways to copy them. For a company to stand out from its competitors, it should continuously develop new competitive advantages [16].

Business Intelligence gives the business the information it needs to perceive and assess changes in the market, competitors, and other regulatory or legal issues. The better the information one has at his disposal to process and exploit, the easier it is for him to utilize human and material resources. The quality of Business Intelligence depends on the ability of the business itself to manage the information. A way to gain and maintain a competitive advantage can be considered the application of Business Intelligence systems by the company. The data available to the business is a valuable, intangible asset, while information is often considered the second most important resource of a business, with the most important resource being human resources. Business Intelligence is characterized by the fact that it provides the right information, to the right user, at the right time and offers the ability for businesses to make quick and effective decisions. Specifically, below is how businesses can take advantage of Business Intelligence to gain a competitive advantage.

### Business intelligence and value chain

Competitive advantage can be emerged or created within a company's value chain. An organization’s cost position reflects the total cost of performing value activities, relative to the corresponding total cost of competitors [17]. Similarly, a firm's ability to differentiate reflects the contribution of each value activity to satisfying buyers’ needs. Many of the company's activities—not just the products and services produced—contribute to differentiation. Buyers’ needs, in turn, depend not only on the products and services of the companies, but also on their other activities, such as after-sales services or logistics. Every value activity includes physical and information processing elements. Physical inputs refer to all the manual tasks required to perform the activity. The elements of information processing include the processes required to record, manage, and channel the necessary data to perform each activity. Also, every value activity creates and uses information. [14]. Information technology is spreading throughout the value chain and so, using Business Intelligence systems, operations are optimized and controlled. Also, close links are created between the different activities within the firm, and companies can now coordinate their actions more closely with those of their buyers and suppliers [13].

Ultimately, the value chain helps an organization determine the “value” its business activities have for its customers. This model emphasizes those activities where business strategies can best be implemented and where information systems such as Business Intelligence are most likely to have a strategic impact. By adding value and thereby creating competitive advantage, Business Intelligence could contribute to every activity in the value chain. Each company, to utilize Business Intelligence systems to obtain a competitive advantage, should identify through the value chain which are those points that need investment in information systems and will make it stand out from the competition through the provision of valuable knowledge.

### Business intelligence and cost and differentiation business strategies

Regards the cost reduction, information technology can change business costs in any activity in the value chain. In the past, technology’s impact on cost reduction was limited to repetitive information processing activities. Today these constraints do not exist and very often information technology changes the cost of activities in such a way that the relative cost position of the firm can be affected. Organizations can use Business Intelligence systems to radically change the cost of their business activities by reducing them [4, 18].

However, as for the enhancement of the differentiation, by using information technology, the personalization of manufactured products is achieved. By using Business Intelligence systems, it is possible to collect data on a company’s customers such as demographic and psychographic data, on their preferences, their purchasing habits, the amount they consume, data which if exploited by the company can create differentiated products that are difficult for competing firms to copy [16]. This information enables companies to make strategic decisions that will result in maximizing their profits. Customer intelligence, or customer analysis, is not only an integral part of maintaining a company’s competitive advantage, but also contributes decisively to the company’s survival. Customer intelligence enables the marketing team to design and implement “smart” offers and programs for the most appropriate customers, using the right distribution channel at the right time every time. This tactic can help the business optimize its interaction with the customer and improve customer satisfaction.

## Materials and methods

Bibliometric analysis is a useful tool, from which researchers can obtain useful information about the current state of research in specific areas of interest. The main utility of bibliometric analysis is both the recording and processing of data related to scientific publications and the extraction of relevant bibliometric indicators. The use of bibliometric indicators concerns the determination of the main characteristics and volume of Greek scientific production in publications [19, 20].

It is worth noting that in recent years there has been a large and continuous increase in the international field, in studies that use the method of bibliometric analysis to conduct results. The use of bibliometric indicators of research activity play an important role in the formation, if possible, of an objectively measurable picture of research systems. In addition, they are used to evaluate research organizations, groups, and researchers. Also, to identify the research fields in which the scientific community is active, to investigate the new research fields that are emerging as well as the scientific networks that are created for the implementation of common research goals. In more detail, given the data collected from scientific publications, information is extracted that identifies the characteristics and trends of research production by institution level, country, or a wider set of countries. An assessment is made of the impact of a scientific project as well as an evaluation of the research activity and finally it is possible to identify national and multinational networks between scientists and scientific disciplines [21].

Finally, bibliometric indicators express the number of publications with numerical data. Regarding the number of publications, it is the simplest indicator to record the production of scientific works and consequently the research work per scientist, organization, scientific discipline, or country. Apart from the number of publications, the analysis of citations to publications from other scientific publications is considered as the most common bibliometric indicator, which is used to assess the impact and originality of the scientific work [22].

### Data sources and collection

Scopus database has been selected, as the main source of data for the current research. The database of Scopus was created in 2004 by Elsevier and is one of the largest “peer reviewed” databases globally, which covers more than 24,000 active academic journal titles in multiple thematic areas of high research interest [23, 24].

Data for the current study were retrieved in September 2022 with the entered search terms to be the following: [(“business intelligence”* OR “business intelligence solutions” OR “business analytics” OR “decision support systems” OR “strategic monitoring”) AND (“small enterprises” OR “medium enterprises” OR “small and medium enterprises”) AND (“competitive advantage”) AND (“decision making process” OR “decision making” OR “management”)]. English was selected as the search language. As it has been referred above, Scopus database includes various types of documents, but only original articles were considered for the current analysis. Finally, a total of 317 documents were selected for consideration.

### Research method

**Fig. 1:**
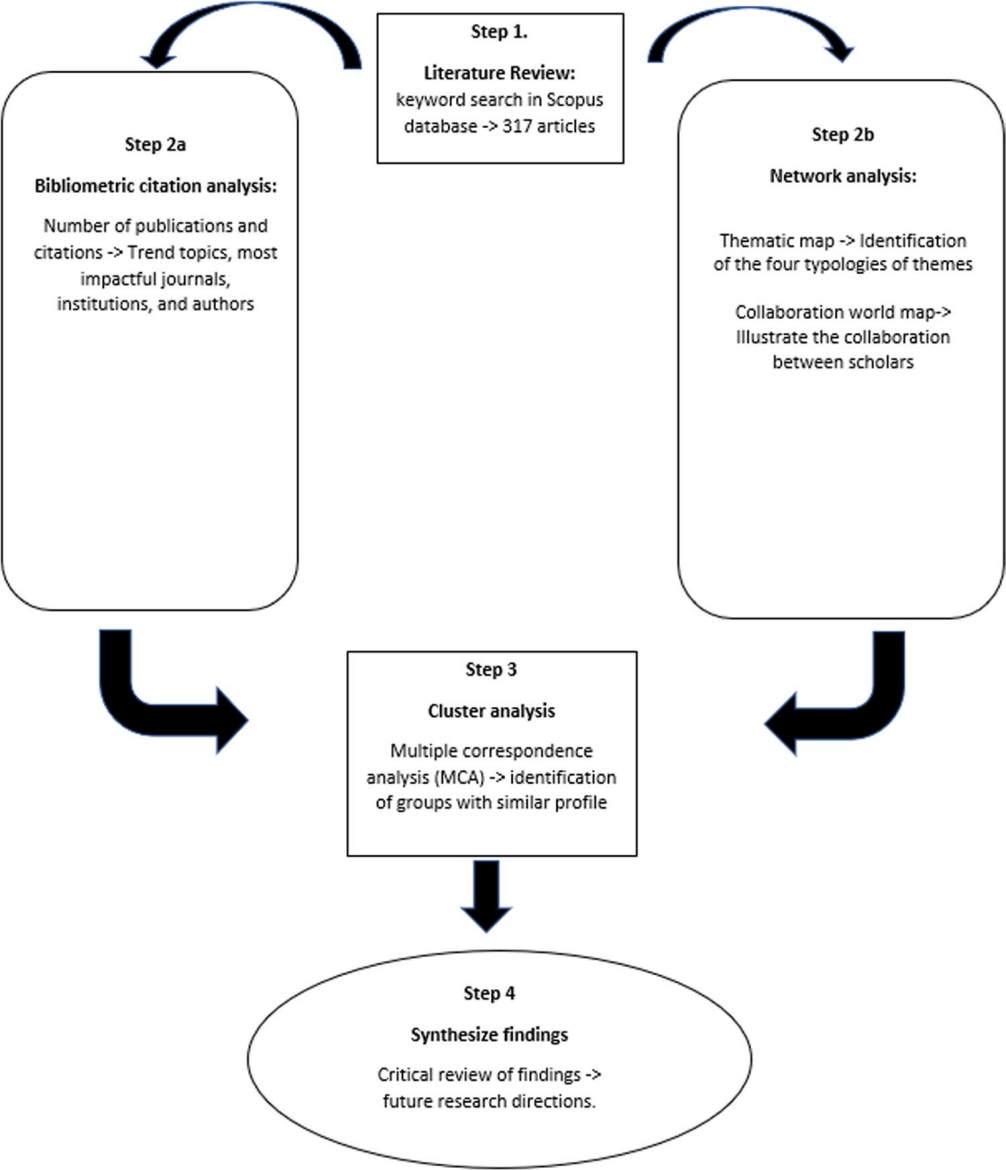
Methodological design for bibliometric process. Source: Own elaboration

The current research may show the bibliometric indicators using both Biblioshiny and VOSviewer, which will emphasize the extent to which the model of business intelligence helps to boosting competitive advantage and increasing the decision-making process. The number of publications and articles, as well as the number of citations and keywords, are some of the primary bibliometric indicators employed in this study endeavor. Additionally, the development of various maps and figures will show the research state and dynamics of the business intelligence model from 2007 to 2022 in order to visualize the findings (Fig. 1).

## Results

Table 1 provides the descriptive information of the collected data that are related to the research trends of business intelligence model and its contribution in the enhancement of competitive advantage and decision-making process of SMEs. The first examination returned 509 documents, whereas the authors’ restriction to the selection of solely articles yielded 317 documents. All these publications use 848 author keywords and 817 keywords plus. The referent period of the analysis is the time between 2007 and 2022. Moreover, the selected papers have written by 809 authors and only 45 have been written by one-single author. In addition, there is a high collaboration in publications in the studied field of business intelligence in SMEs, which can be approved by the collaboration index. Specifically, document per author ratio is 0.392, which means, on average, almost three authors have written one document.

**Table 1:** Descriptive characteristics of literature regards business intelligence in SMEs. Source: Scopus/Biblioshiny

Description	Results
Documents	317
Sources (Journals)	246
Author’s keywords (DE)	848
Keywords plus (ID)	817
Timespan	2007:2022
Average citations per documents	12.55
Authors	809
Author appearances	898
Authors of single-authored documents	45
Authors of multi-authored documents	764
Single-authored documents	57
Documents per Author	0.392
Authors per document	2.55
Co-Authors per documents	2.83
Collaboration index	2.94

**Fig. 2:**
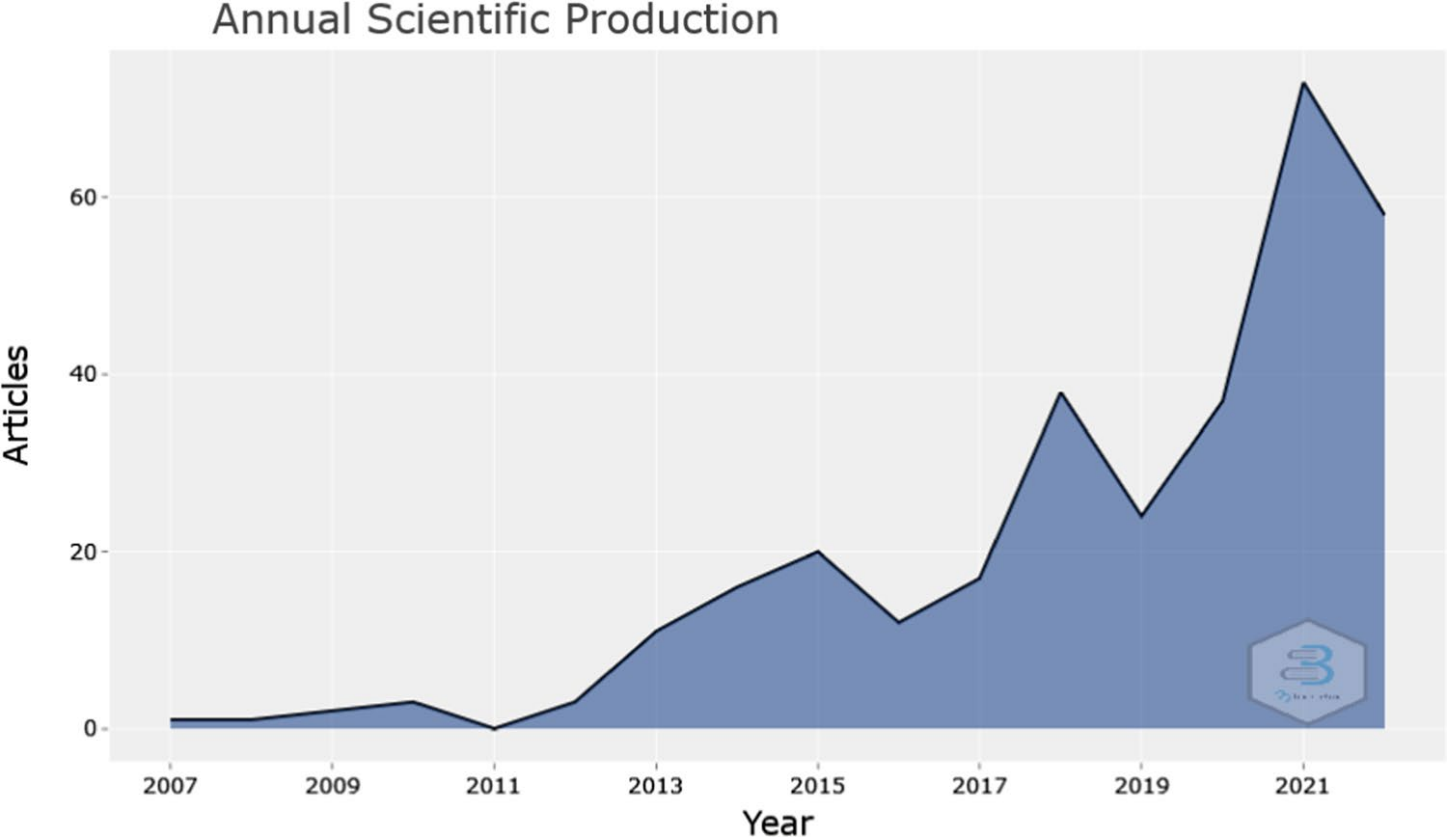
Annual scientific production. Source: Scopus/Biblioshiny

**Fig. 3:**
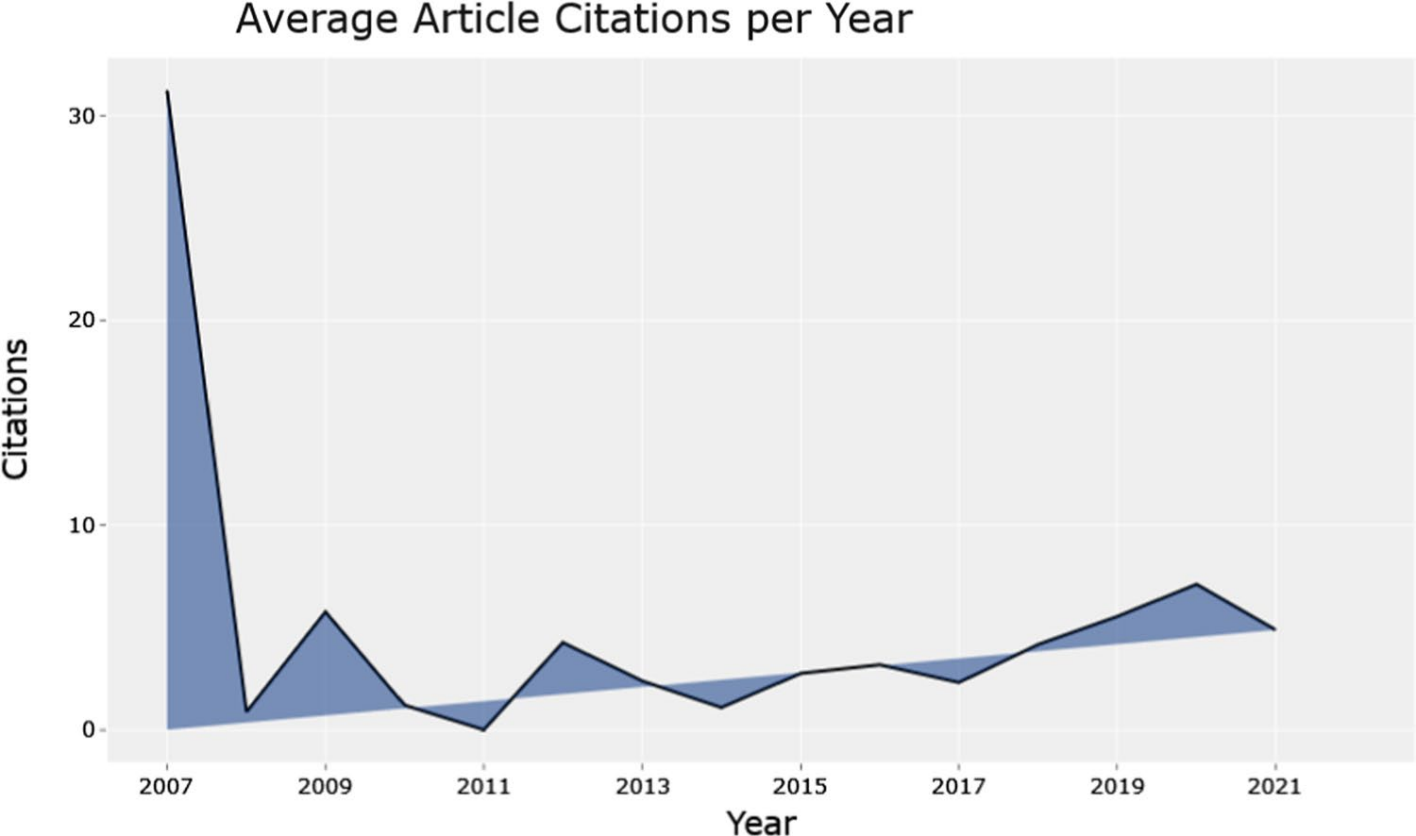
Average articles citation per year. Source: Scopus/Biblioshiny

Figure 2 presents the annual scientific production, while Fig. 3 illustrates the citation per year for publications related to business intelligence in SMEs and the way that this model affects their competitive advantage and decision-making process. At the beginning of the research year there is a limited production of scientific papers (Fig. 2), which indicates the restricted knowledge of SMEs regards the model of business intelligence [25]. It is noteworthy that between 2009 and 2010 there was a little development on the scientific production in the studied field, but 1 year later (2011) there is a sharp decrease in the production of papers, which is based on the financial crisis that affects the global community. However, after 2013, there was a fluctuation trend in the publications and citations of studies in business intelligence and SMEs, while 2021 can be characterized as the peak year of both publications and citations. This increase is due to the outbreak of Covid-19, which enhanced more the role of emerging technologies in the business arena [26, 27]. The transition to the new digital age helps small and medium-sized enterprises to create a new digital network, a factor that is crucial for such enterprises to respond to the increased competition, to promote their products in larger markets and to implement new innovative proposals with greater success rates. Thus, SMEs can increase their competitive advantage [4]. Additionally, regarding Fig. 3, the development of the typical article's citation shows a distinct tendency. This is predicated on the assumption that when a new body of research arises, it takes time for it to develop, and as more papers in that body of literature are published, the number of citations increases as well.

### Bibliometric citation analysis

Table 2 ranks the most influential journals in the studied field of business intelligence in SMEs. Journals have been ranked based on the number of the published papers that are related to the research field. “Sustainability” was the journal with the highest number of published articles on business intelligence and SMEs (15) during the period 2007–2022. “International Journal of Business Information Systems”, “Journal of Information and Knowledge Management” and Journal of Knowledge Management” are ranked in the second position with 5 articles each. Moreover, most of the cited journals on the field of business intelligence are indexed by Scimago and ABS list, while plenty of them are subject to the research area of Information System and Management.

**Table 2:** Top 20 journals in the field of business intelligence in SMEs. Source: Scopus/Biblioshiny

Ranking	Sources	Number of publications	Subject area	h-Index	Ranking by ABS list	Ranking by Scimago list
1	Sustainability (Switzerland)	15	Energy Engineering and Power Technology	109		Q1
2	International Journal of Business Information Systems	5	Information Systems and Management	28	1*	Q2
3	Journal Of Information and Knowledge Management	5	Computer Science Applications	22		Q3
4	Journal Of Knowledge Management	5	Management of Technology and Innovation	124	1*	Q1
5	Information Systems Management	4	Computer Science Applications	61	2**	Q1
6	Technological Forecasting and Social Change	4	Management of Technology and Innovation	134	3***	Q1
7	Benchmarking	3	Strategy and Management	66	1*	Q1
8	Global Business Expansion: Concepts Methodologies Tools and Applications	3	Information Systems and Management			
9	Industrial Marketing Management	3	Business, Management and Accounting	147	3***	Q1
10	International Journal of Business Intelligence And Data Mining	3	Information Systems and Management	20		Q4
11	International Journal of Information Management	3	Information Systems and Management	132	2**	Q1
12	Journal Of Enterprise Information Management	3	Management of Technology and Innovation	67	2**	Q1
13	Advances In Intelligent Systems and Computing	2	Computer Science (miscellaneous)	48		Q4
14	Annals Of Operations Research	2	Management Science and Operations Research	111	3***	Q1
15	Applied Sciences (Switzerland)	2	Computer Science Applications	75		Q2
16	Asia Pacific Journal of Marketing And Logistics	2	Strategy and Management	51	1*	Q1
17	Business Process Management Journal	2	Business and International Management	87	2**	Q1
18	Contributions To Management Science	2	Management of Technology and Innovation	15		Q4
19	IFIP Advances in Information And Communication Technology	2	Information Systems and Management	56		Q3
20	Industrial Management and Data Systems	2	Management Information Systems	109	2**	Q1

Table 3 presents the most related publications regards the role of business intelligence in SMEs and the contribution of this business model in the enhancement of the competitiveness and improvement of the decision-making process of SMEs. The list of Table 3 includes the twenty most relevant articles to the subject studied in this article. At the top of the list is the publication of Maier, who published the first study in 2007 on the role of information analytics that has transformed businesses, organizations and society [28]. Also, the aim of Nguyen’s research was to gain a clearer understanding of Information Technology (IT) adoption in SMEs by analyzing and contrasting the current literature. Whilst describing how and why SMEs acquire IT, this paper was aiming to present the factors that influence the adoption process both positively or negatively [2]. In addition, Chatterjee et al.in their paper are referred to the contribution of the Industry 4.0 era, which has brought a breakthrough in emerging technologies in the fields such as artificial intelligence, machine learning, deep learning, robotics, the Internet of Things, fully autonomous vehicles, 3D printing and much more [29]. Also, authors in their study have attempted to identify social, environmental, and technological factors that affect the integration of artificial intelligence embedded technology by digital manufacturing and production organizations. Thus, the framework of technology-organization-environment (TOE) is used to explore the applicability of Industry 4.0. Furthermore, TOE model is indicated as one of the most important in helping SMEs to integrate emerging technologies in their business model [29].

**Table 3:** Most globally cited articles. Source: Scopus/Biblioshiny

Paper	Total Citations	TC per Year	Normalized TC
Knowledge management systems: information and communication technologies for knowledge management	469	29,312	1
Harvesting big data to enhance supply chain innovation capabilities: an analytic infrastructure based on deduction graph	265	33,125	13,7306
Proactive CSR: An Empirical Analysis of the Role of its Economic, Social and Environmental Dimensions on the Association between Capabilities and Performance	191	19,1	8,9025
Ranking of drivers for integrated lean-green manufacturing for Indian manufacturing SMEs	166	33,2	9,9495
Social media and entrepreneurship research: a literature review	130	43,333	9,1271
Information technology adoption in SMEs: an integrated framework	126	9	1,68
Critical Success Factors for Implementing Business Intelligence Systems in Small and Medium Enterprises on the Example of Upper Silesia, Poland	121	11	2,8359
Role of big data management in enhancing big data decision-making capability and quality among Chinese firms: a dynamic capabilities view	97	24,25	5,8346
Toward Better Understanding and Use of Business Intelligence in Organizations	86	12,286	4,5066
Two decades of research on business intelligence system adoption, utilization and success—a systematic literature review	81	20,25	4,8722
Big data analytics adoption: determinants and performances among small to medium-sized enterprises	69	23	4,8444
Intellectual capital and performance measurement systems in Iran	69	13,8	4,1356
Big Data-Savvy Teams’ Skills, Big Data-Driven Actions and Business Performance	56	14	3,3684
A linear regression approach to evaluate the green supply chain management impact on industrial organizational performance	55	11	3,2965
An investigation of key competitiveness indicators and drivers of full-service airlines using Delphi and AHP techniques	52	7,429	2,7249
Agile supply chain management: where did it come from and where will it go in the era of digital transformation?	49	16,333	3,4402
Understanding AI adoption in manufacturing and production firms using an integrated TAM-TOE model	48	24	9,8151
A problem-solving ontology for human-centered cyber physical production systems	47	9,4	2,817
An information sharing theory perspective on willingness to share information in supply chains	46	7,667	3,9695
Evaluating the Drivers to Information and Communication Technology for Effective Sustainability Initiatives in Supply Chains	43	8,6	2,5773

Table 4 presents the most related affiliations in the studied field of business intelligence in SMEs. The Universiti Sains Malaysia (USM) is positioned first and is among the best business schools globally. Moreover, Universiti Kebangsaan Malaysia is one of the business schools with plenty of research years in strategic planning, decision-making and control which is crucial for business in achieving competitive advantage. In the third position is ranked the Suan Sunandha Rajabhat University.

**Table 4:** Most relevant affiliations. Source: Scopus/Biblioshiny

Affiliations	Articles
Universiti Sains Malaysia	15
Universiti Kebangsaan Malaysia	12
Suan Sunandha Rajabhat University	11
Universiti Teknologi Malaysia	11
Bina Nusantara University	9
Abu Dhabi University	8
University of Ljubljana	7
Edith Cowan University	6
Norwegian University of Science and Technology	6
Nove De Julho University	6
Tshwane University of Technology	6
Dalian University of Technology	5
Federal University of Santa Maria	5
Instituto Politécnico Nacional-Unidad Profesional Interdisciplinaria De Ingeniería Y Ciencias Sociales Y Administrativas	5
King Faisal University	5
Mci Entrepreneurial School	5
National Kaohsiung University of Science and Technology	5
Universiti Malaysia Sabah	5
Universiti Putra Malaysia	5
University Of Science and Technology of China	5

**Fig. 4:**
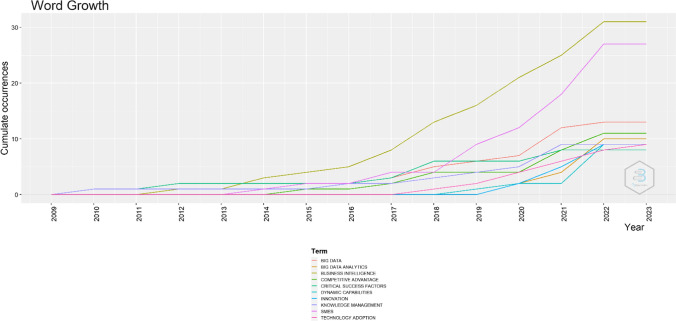
Word growth overtime. Source: Scopus/Biblioshiny

**Fig. 5:**
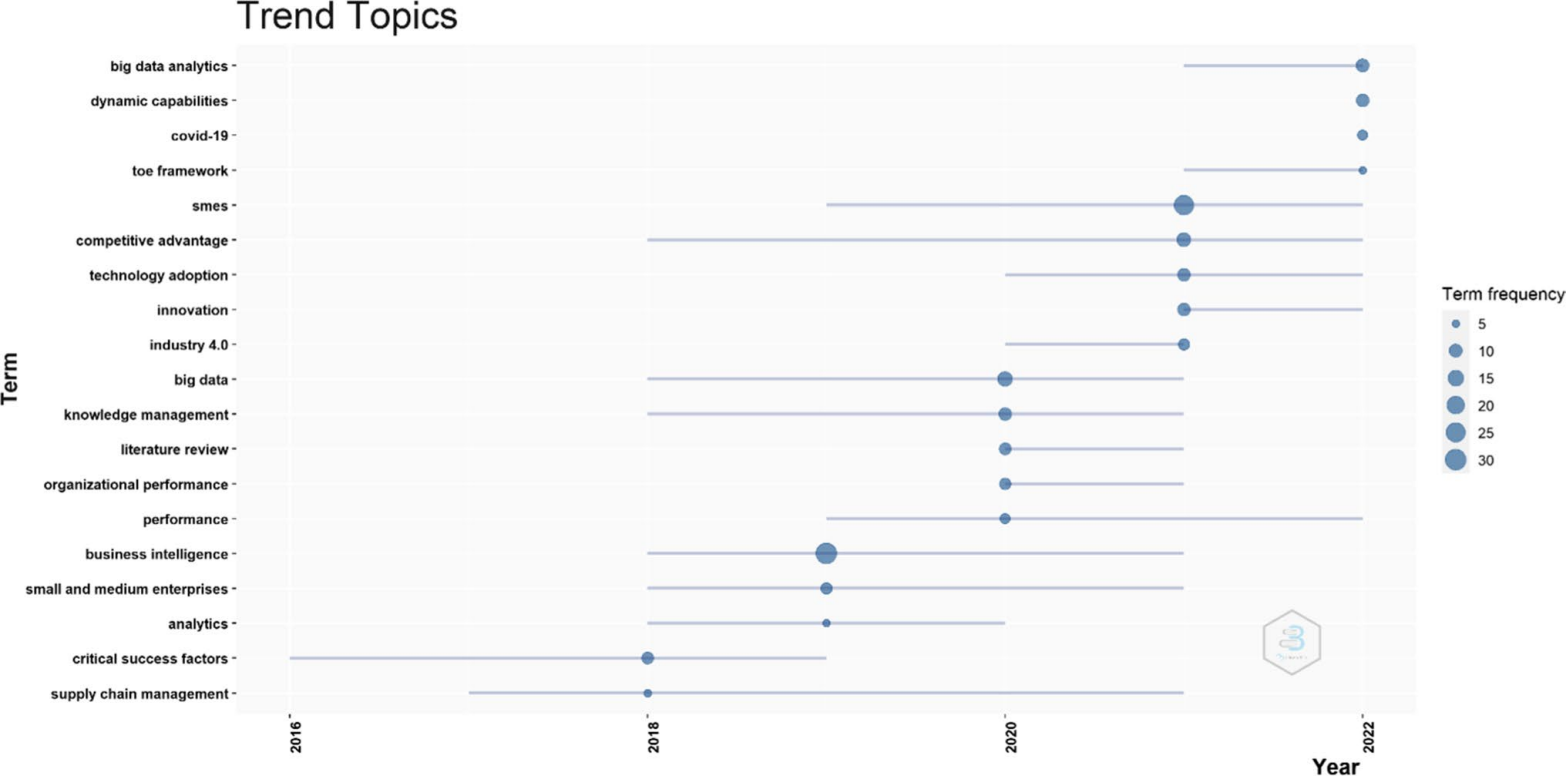
Research trends on the field of business intelligence in SMEs

In addition to the most relevant journals, publications and affiliations, Fig. 4 presents the word growth in literature thought the years. As shown in the figure, the keywords of business intelligence in SMEs have started to grow after 2013 more regularly, while the sharp increase for both keywords is illustrated in the year 2019 and after. This is based on the outbreak of Covid-19, which hit hard small and medium businesses. Thus, business intelligence model has been called to mitigate these negative effects, reduce the bureaucracy that burdens SMEs and strengthen their resilience by enhancing their competitive advantage. Moreover, the above are presented in Fig. 5 too, which illustrates the research trend topics of the studied field.

### Network analysis

**Fig. 6:**
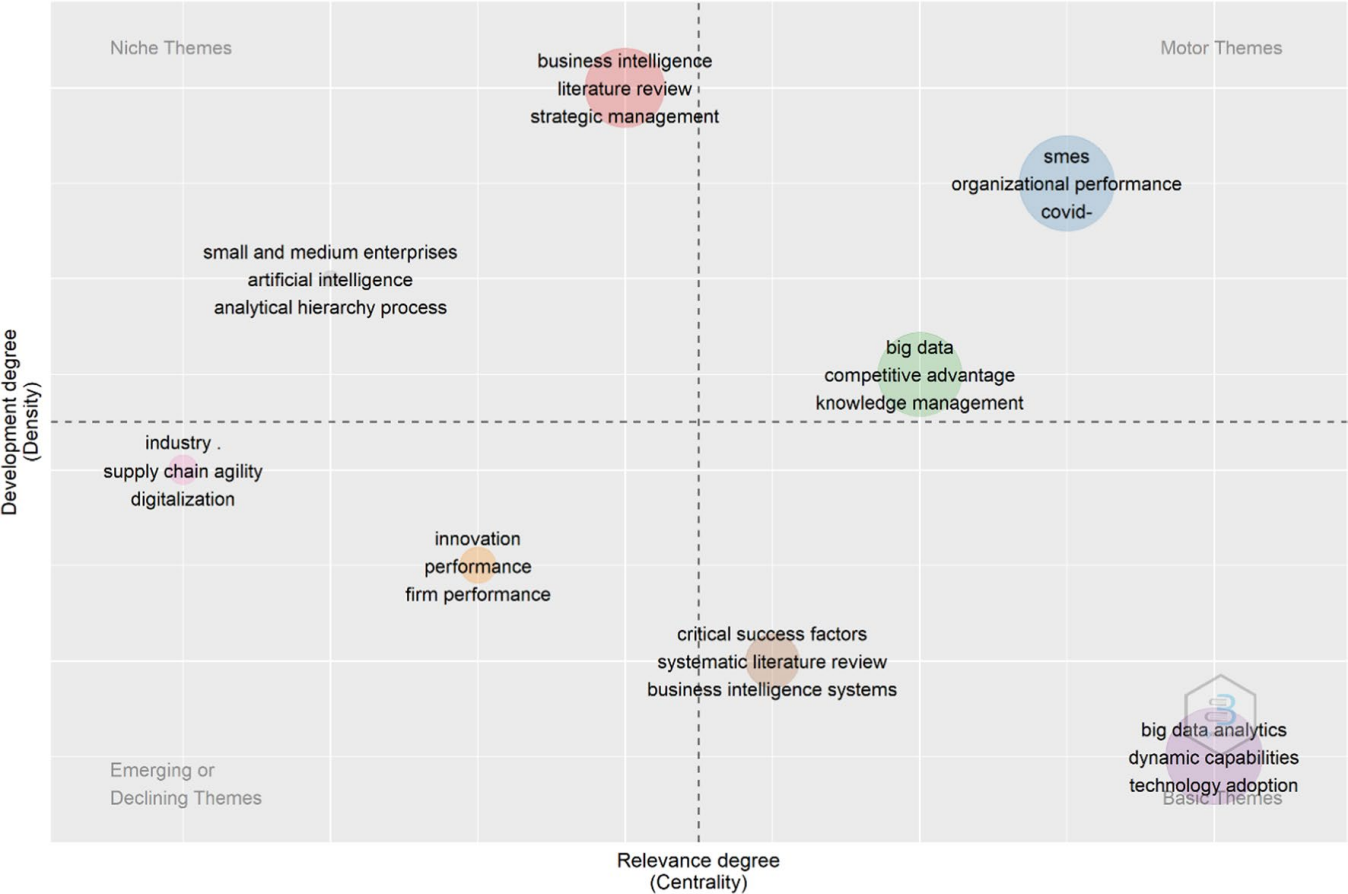
Thematic map. Source: Scopus/Biblioshiny

To detect the main research themes in the studied field, thematic map has been developed with the use of Biblioshiny. Thematic map apart from the axis of centrality, which presents the importance of each of the entitled theme, and the axis of density, which represents the development of the theme that has been chosen. The map is divided into four quadrants, which each of them illustrates a different theme category. The quadrant that is positioned in the lower left quadrant represents the emerging or declining themes, which are new themes that can emerge or drop from the research area. The quadrant that is positioned in the lower right side of the thematic map indicates the basic or transversal themes and are characterized by low density and high centrality. Issues that are included in this part of the map illustrates that much research has been done on these. Moreover, the quadrant that is in the upper left part of the map are characterized by high density and low centrality and are called as niche themes. Finally, the quadrant in the upper right part of the thematic map is represented by both high density and high centrality. These themes are characterized as motor themes, which are developed and crucial [21, 22]. Thus, Fig. 6 illustrates the thematic map for the integration of business intelligence in SMEs.

### Cluster analysis

**Fig. 7:**
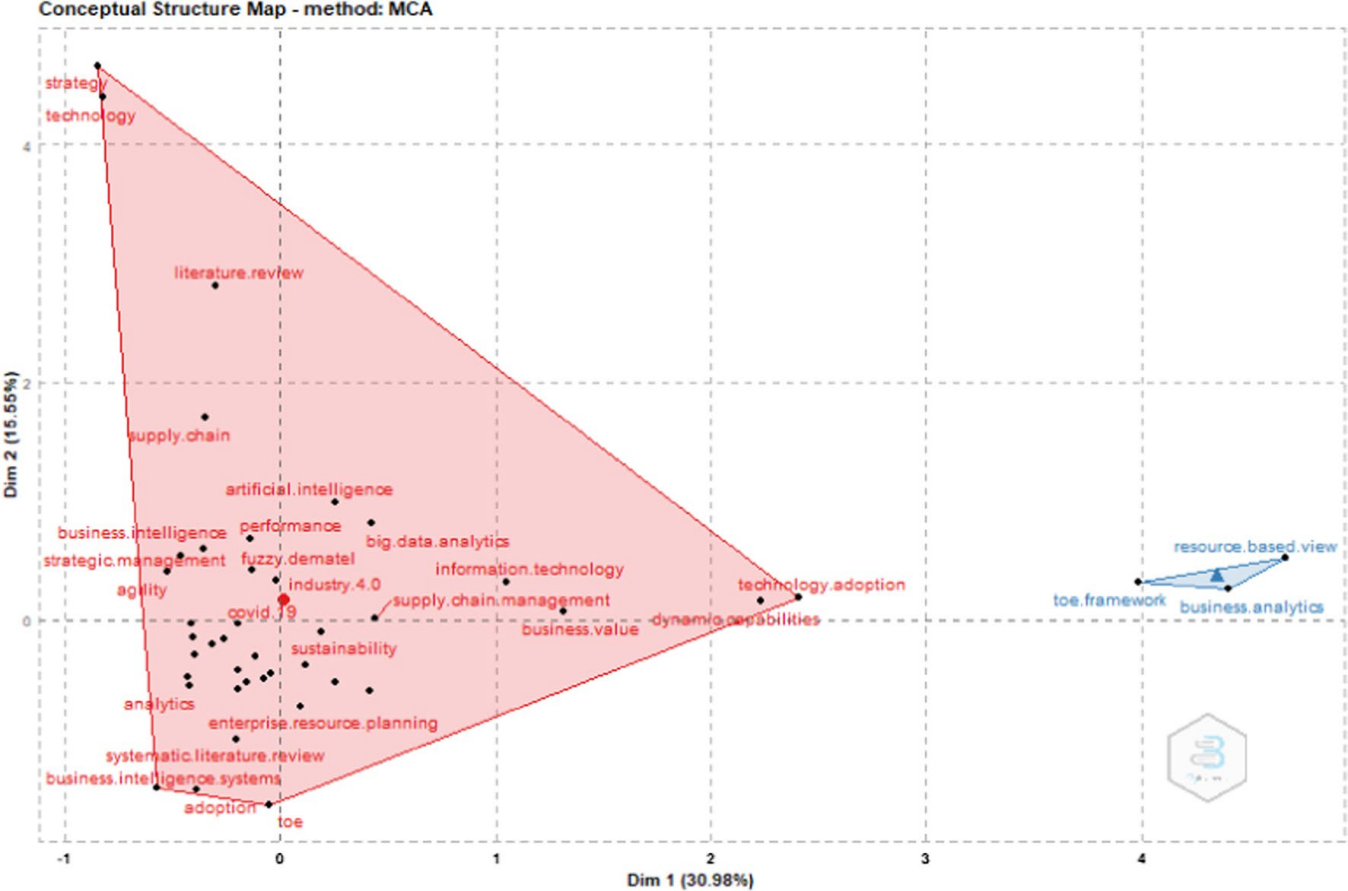
Cluster analysis based on the MCA method. Source: Scopus/Biblioshiny

Although SMEs have started integrating business intelligence model to enhance their competitive advantage and improve the decision-making process, Multiple Correspondence Analysis (MCA) (Fig. 7) reveals that business intelligence should be combined with traditional business models that help especially SMEs to integrate emerging technologies in their operational system. TOE framework is among those business models that can facilitate SMEs with the adoption of business analytics [7]. The technology-organization-environment framework, also known as the TOE framework, is a theoretical framework that explains the adoption of technology in organizations and describes how the process of adopting and implementing technological innovations is influenced by the technological, organizational, and environmental context. Although the spread of the coronavirus has acted as an opportunity for many companies to accelerate their digital transformation, SMEs, which represent a large percentage of the global economy, are slow to adopt new technologies, which has a negative effect on their competitiveness [30–32]. Therefore, the development of a new business model, which will be based on the TOE and business intelligence is crucial for SMEs to adopt emerging technologies and enhance their competitive advantage [30, 33].

## Discussion and conclusions

The modern business has access to a vast amount of data that comes from its clients, suppliers, internal operations, and other external sources. With the right processing, this data can be transformed into helpful information that can be shared across all company departments, improving communication, and helping to plan the organization's various processes. It can also make a significant contribution to decision-making at all organizational levels. Making decisions is essential for every business' survival and growth in the cutthroat business world of today. Business intelligence and its numerous tools are at the heart of this problem.

Business Intelligence is a technology that includes applications and methods for the analysis and processing of data, and aims to discover knowledge, this knowledge should be used by management to make decisions. Decision-making by a Business Intelligence system includes several stages, such as the collection of data from the various information systems available to the company, the storage, retrieval and sorting of the appropriate data as well as their processing and transformation into useful information. For all these activities the European Union (EU) has a wealth of tools such as On-line Analytical Processing, data warehouses, data centers, visualization, and many other tools. BI systems contribute to decision-making at all levels of an enterprise, from the daily processes of the operational level to the important and long-term decisions made by management at a strategic level, in contrast to the rest of the information systems that can have a business that usually serves processes of a certain level. While initially Business Intelligence systems were only used by large multinational organizations due to their high costs, today these systems are used in almost all business sectors and especially in industrial and commercial companies, insurance companies, banks, and telecommunications as it has proven in practice that their benefits far outweigh their costs. The advantages of using them are many and varied, the most important ones being a better understanding of customers and their needs, reducing costs and failures and creating a competitive advantage over other businesses in the industry.

It is important to mention that choosing an EU system is not a simple matter and should be done after careful research on the part of businesses, so that the product they choose serves their needs and can perform the simplest to the most complex functions. It should primarily be easy to use, so that specialized personnel and capabilities are not required adapting the system to new data. It is also necessary to allow easy sharing of data between users. In addition, the integration of many different databases should be easy, and finally not required expensive hardware for its operation. Technological developments, the integration of markets at a global level, the financial crisis and constant changes have significantly intensified the competition between businesses, which must meet the needs and desires of consumers. To achieve this, it is necessary to have coordination between all the processes of a company, as well as correct information between departments.

This study highlighted the importance of Business Intelligence systems in the enhancement of the competitive advantage and the improvement of decision-making process of SMEs. Adopting BI across the organization requires a thorough understanding of what it is, why it is used, and the associated advantages. Although several research looked at BI adoption drivers and theories, there aren’t enough studies to fully understand how ready SMEs are to implement BI. Owner or senior managers of SMEs may find this information useful in their efforts to promote BI in a more proactive manner. It would also be helpful to conduct more empirical research on the factors and obstacles that affect BI adoption. To help in identifying the elements that contribute to effective BI deployment, a few frameworks and models have evolved. Thus, the need of a business model that will combine both Business Intelligence and TOE framework is crucial [34]. The analysis indicated TOE framework as very important for SMEs, because it facilitates to the integration of emerging technologies by SMEs. Therefore, executives should focus on that issue and develop a business model based on the above factors [30]. Once the new business model will be developed then the next step is to be applied to a sample of SMEs from different economic sectors. This can be achieved by researchers through empirical research, which will be based on questionnaires that will be designed for this purpose. Findings may be provided in a research paper to assist SMEs adopt this business model correctly and to understand the advantages and disadvantages of it.

Additionally, SMEs should strengthen their foundations for implementing and using business intelligence (BI) methods to enhance their business growth. The SMEs engaged in the initiative make use of BI-knowledge by setting it to use in building digital and circular economy-based goods, services, processes, or activities. SMEs can achieve that by employing collaborative, innovative, and agile practices, and techniques. The SMEs are concentrating on utilizing marketing automation and digital-based chances while establishing commercialization operations. In addition, there should be more cooperation between SMEs and institutes of higher learning. To share and improve the business knowledge-based solutions of SMEs, benchmarking workshops and meetings can be organized. College and university students can take a proactive role in the growth of SMEs.

In this rapidly changing and evolving environment, consumers now demand faster and more efficient service from businesses. To remain competitive, businesses and especially SMEs should meet or, better yet, exceed consumer expectations. Thus, SMEs will have to rely more heavily on Business Intelligence systems to stay ahead of trends and developments in events. Business Intelligence users are beginning to demand real-time analysis of their activities, especially those engaged in "frontline" activities [3, 35, 36]. They monitor developments and constantly look for new and up-to-date information in the same way that they monitor stock prices online. Monthly and even weekly analyzes will not be enough for them. In the following years, SMEs will depend on real-time business information in the same way that people search for information online with just a “click”. Also, the upcoming period, business information will become more democratized as any employee within an organization will be able to access the information to be informed about the performance of their department. In conclusion, in the future the required Business Intelligence capabilities will increase, at the same rate as consumer expectations and demands. Therefore, it is imperative for businesses to improve their pace to remain competitive.
